# Association of acylcarnitine species and diabetes incidence in a population-based apparently healthy cohort

**DOI:** 10.1186/s40842-026-00298-0

**Published:** 2026-06-01

**Authors:** Ko Ko Maung, Rebecca Borreggine, Hector Gallart-Ayala, Julijana Ivanisevic, Pedro Marques-Vidal

**Affiliations:** 1 Department of Medicine, Internal Medicine, Lausanne University Hospital (CHUV), University of Lausanne, Office BH10-642, Rue du Bugnon 46, Lausanne, 1011 Switzerland; 2 Metabolomics & Lipidomics Platform, Faculty of Biology and Medicine, University of Lausanne, Lausanne, Switzerland

**Keywords:** Acylcarnitine, Diabetes mellitus, Risk scores, Biomarkers, Prospective studies

## Abstract

**Background:**

Current diabetes risk scores often fail to detect early metabolic changes. Acylcarnitines species (ACs), intermediates of mitochondrial fatty acid oxidation, may indicate early dysregulation in diabetes, but prospective evidence is limited. We investigated the associations between circulating ACs and 10-year incidence of diabetes, and whether AC improve the predictive performance of established diabetes risk scores.

**Methods:**

Data from 1,976 apparently healthy adults in a population-based cohort. Incident diabetes was defined as HbA1c ≥ 48 mmol/mol or initiation of glucose-lowering medication at follow-up. Associations were analysed using bivariate and multivariate models, and predictive value was assessed by adding ACs individually to FINDRISC, SDA, Kahn clinic, Kahn clinic+biologic, Wilson, Balkau and Griffin scores.

**Results:**

Over 10 years, 35 participants developed diabetes. Higher baseline levels of Propionyl-, Isovaleryl-, Hexanoyl-, and Octenoylcarnitine were associated with incident diabetes in bivariate analysis whereas only Propionylcarnitine remained associated in multivariate analyses, mean ± sem 376.3 ± 2.7 vs. 419.1 ± 20.4 nmol/L in non-Diabetes and incident diabetes, respectively, *p* < 0.05. Sensitivity analyses using both diabetes and prediabetes incidence showed similar pattern of associations compared to the primary analysis. In predictive analyses, Propionyl- and Isovalerylcarnitine remained independently associated with incident diabetes after adjustment for each established diabetes risk scores. Adding Isovalerylcarnitine to the risk scores improved discrimination in three out of seven risk scores: AUC 0.785 (0.710–0.859), 0.841 (0.789–0.892), and 0.841 (0.789–0.892) for Balkau, SDA and FINRISK, respectively.

**Conclusion:**

Specific AC species, particularly Propionyl- and Isovalerylcarnitine, predicted 10-year risk of diabetes and improved predictive performance beyond established risk scores, suggesting their potential as early metabolic markers.

**Supplementary Information:**

The online version contains supplementary material available at 10.1186/s40842-026-00298-0.

## Research insights

- What is currently known about this topic? (max. 3 highlights)
  - Current diabetes risk scores heavily rely on the traditional markers.
  - The traditional diabetes biomarkers often fail to detect early metabolic changes.
- What is the key research question? (formatted as a question)
  - Do acylcarnitine (AC) levels improve incident diabetes prediction beyond current risk scores?
- What is new?
  - Higher level of a specific short-chained AC (SCAC) was associated with 10-year diabetes incidence.
  - SCACs provided additional predictive value for diabetes incidence beyond traditional risk scores.
  - Inclusion of selected SCACs to the risk scores improved the discrimination.
- How might this study influence clinical practice?
  - SCAC may serve as early metabolic markers that could enhance diabetes risk prediction.

**Supplementary Information:**

The online version contains supplementary material available at 10.1186/s40842-026-00298-0.

## Introduction

Globally, diabetes affected over 589 million people in 2024, and is projected to rise to 900 million by 2050 [1]. In 2021, there were estimated 1.6 million diabetes-related deaths, among which seven out of ten occurred prematurely [2]. In addition, even in a high-income country like Switzerland, known for having one of the best healthcare systems in Europe, more than half of the diabetes patients receiving treatment presented with suboptimal control [3]. Albeit the rising global burden of diabetes, nearly half of the people with diabetes were unaware of their diabetes condition [4]. Therefore, early diagnosis of diabetes is crucial, especially in the context where early lifestyle and pharmacological interventions can significantly reduce both incidence and all-cause mortality of pre-diabetic patients [5].

Current screening strategies for diabetes, including most predictive risk scores, heavily rely on the traditional biomarkers for diabetes, such as fasting plasma glucose (FPG) and HbA_1_c [6]. While these biomarkers have been widely used for diagnosing and monitoring diabetes, they have major limitations in detecting early metabolic changes in the early stages of diabetes [7]. By the time patients present to the clinic, some of the chronic diabetes-related macro and/or microvascular complications, such as cardiovascular, kidney, ophthalmic and neurological complications, have already developed [8].

Since diabetes is one of the metabolic disorders, recent studies have adapted metabolomic approach for identifying novel biomarkers, leading to promising novel diabetes markers [7]. One of the promising candidates is acylcarnitines (AC): esters formed by the conjugation of L-carnitine with acyl groups. ACs are derived from either fatty acid or amino acid metabolism, essential intermediates in cellular energy metabolism, particularly in β-oxidation [9]. ACs, especially the long-chain Acylcarnitine (LCAC), are suggested to be associated with insulin resistance by decreasing the phosphorylation of the insulin receptor through increased activity of protein tyrosine phosphatase 1B (PTP1B) and suppression of Protein kinase B (PKB), also known as Akt [10]. Several cross-sectional studies suggested a positive association between short-chain Acylcarnitine (SCAC) (C2:0 and C3:0), medium-chain Acylcarnitine (MCAC) (C8:0 and C10:0), and LCAC (C16:0 and C18:0) and T2DM. In contrast, one study showed a negative association of SCAC C3:0 with T2DM [9]. In a case-cohort study of 663 Spanish participants, plasma LCAC (C14:1, C16:0 and C18:1) predicted the development of T2DM beyond traditional risk factors [11]. Nevertheless, research findings regarding the association of ACs with T2DM are inconsistent, except perhaps for the positive association between long-chain ACs and T2DM.

Therefore, we aim to investigate the associations of circulating AC species and the incidence of diabetes, and the predictive capacity of baseline circulating AC species on the diabetes incidence in a population-based, apparently healthy sample.

## Participants and methods

### Participants

The study used data from the CoLaus|PsyCoLaus study (www.colaus-psycolaus.ch), a prospective study conducted in the apparently healthy, community-dwelling population of Lausanne, Switzerland. Recruitment began in 2003 and ended in 2006 and included 6,733 participants. The follow-ups were conducted in 2009–2012, 2014–2017 and 2018–2021 with initial5064 participants (75.2%). As the assessment of AC was conducted in the first follow-up, which was therefore considered the baseline of the present analysis. As the initial aim of the study was to establish reference values for healthy people, participants were eligible if their BMI was < 30 kg/m^2^ and they had no diabetes or underlying cardiovascular diseases. Participants taking statins were not eligible for the study.

### Acylcarnitine measurements

#### Sample and calibration curve preparation

For absolute quantification of AC, samples were prepared by adding 250 µL of the diluted (1/500) stock IS mixture of AC in methanol to plasma (20 µL) as described previously [12]. This solution was completed to 300 µL with 0.1% formic acid in water. Samples were then vortexed and centrifuged for 15 min at 4 °C and 2700 g. The resulting supernatant was transferred to LC-MS vials and injected into the Liquid Chromatography-High Resolution Mass Spectrometry Analysis (LC-HRMS) system. Ten-point calibration curves were prepared following the same procedure as samples.

#### Liquid chromatography-high resolution mass spectrometry analysis

A Vanquish Horizon (Thermo Fisher Scientific) ultrahigh-performance liquid chromatography (UHPLC) system coupled to Q-Exactive Focus interfaced with the heated electrospray ionization (HESI) source was used for the profiling of AC. The separation was carried out using a BEH Amide column (1.7 μm, 100 mm × 2.1 mm i.d.) (Waters, Massachusetts, U.S.A.). The mobile phase was composed of A = 20 mM ammonium formate and 0.1% formic acid in water and B = 0.1% formic acid in acetonitrile (ACN). A gradient elution from 95% B (0–2 min) to 65% B (14 min) reaching 50% B at 16 min was applied and followed by 4 min post run for column re-equilibration. The flow rate was 400 µL/min, the column temperature was 25 °C, and the sample injection volume was 2 µL. HESI source conditions operating in positive mode were set as follows: sheath gas flow at 60, aux gas flow rate at 20, sweep gas flow rate at 2,spray voltage at + 3 kV, capillary temperature at 300 °C, s-lens RF level at 60, and aux gas heater temperature at 300 °C. Full-scan HRMS acquisition mode (m/z 50 − 750) was used with the following MS acquisition parameters: mass resolving power at 70,000 FWHM, 1 µscan, 1e6 AGC, and 100 ms as maximum inject time.

The AC species analysed in this study were classified into short-chain (SCAC), medium-chain (MCAC) and long-chain (LCAC) as indicated in supplementary Table 1.

#### Diabetes

Incident diabetes was defined as a glycated haemoglobin (HbA1c) ≥ 48 mmol/mol or initiation of glucose-lowering medication at follow-up. For blood sample collection, venous blood samples (50 mL) were drawn in the fasting state. Biological assays were performed at the clinical laboratory of the Lausanne university hospital within two hours of blood collection. Glucose was assessed by glucose dehydrogenase with a maximum inter- and intra-assay CV of 2.1% and 1.0%, respectively. Glycated haemoglobin was measured by high performance liquid chromatography (HPCL) using the Bio-Rad, D-10TM system (Bio-Rad Laboratories AG, CH-4153 Reinach, Switzerland), with a measurement range of 3.8% (at 18 mmol/mol) to 18.5% (at 179 mmol/mol).

#### Other covariates

Lifestyle data was self-reported. Smoking status was categorized as never, former and current. Participants were asked if they were following any type of diet (to reduce, low in lipids, other…) and the answers were grouped into a single variable categorized as yes (presence of any type of diet) or no. Alcohol consumption was categorized as present or absent. Physical activity was assessed by a questionnaire validated in the population of Geneva [21]. This questionnaire assesses the type and duration of 70 kinds of (non)professional activities and sports during the previous week. Sedentary status was defined as spending over 90% of the daily energy in activities below moderate- and high-intensity, defined as requiring at least 4 times the basal metabolic rate [22].

Waist circumference was measured mid-way between the lowest rib and the iliac crest using a non-stretchable tape and the average of two measurements was taken. Abdominal obesity was defined as a waist circumference > 102 cm (men) or > 88 cm (women).

Blood pressure (BP) was measured thrice using an Omron^®^ HEM-907 (Matsusaka, Japan) automated oscillometric sphygmomanometer after at least a 10-minute rest in a seated position. Different-sized cuffs were available to take into account arm circumference, and the average of the last two measurements was used.

#### Diabetes risk scores

Seven T2DM risk scores were considered: (1) the FINDRISC score [13]; (2) the Swiss Diabetes Association Score [14]; (3) the clinical and clinical-biological scores by Kahn et al., respectively [15]; (4) and (5) the clinico-biological risk score by Wilson et al. [16]; (6) the clinical risk score by Balkau et al. [17], and (7) the clinical risk score by Griffin et al. [18]. The details of each score are summarized in Reference [19] and Supplementary Table 6.

The FINDRISC score was derived from the 10-year follow-up FINRISK study (a Finnish population survey on risk factors on chronic noncommunicable diseases), which consists of 4435 participants and seven variables. The SDAS risk score is adapted from the FINDRISC score, using familial history of diabetes as an additional variable. The risk scores by Kahn et al. were derived in a cohort of 15 792 adults followed up on for 10 years. The clinical risk score (C) consists of nine variables, while the clinical-biological risk score (CB) has four additional biological markers (glucose, triglycerides, high density lipoprotein, uric acid). The clinical-biological risk score of Wilson et al. was derived from the Framingham Offspring Study, where 3140 participants were followed-up on for 8 years; the score consists of six variables among which are three biological ones: glucose, triglycerides, and high-density lipoprotein. The clinical risk score of Balkau et al. was derived from the Data from the Epidemiological Study on the Insulin Resistance Syndrome (DESIR) cohort, where 3817 participants were followed for nine years; it consists of four variables. Finally, the score of Griffin et al. was derived from a cross-sectional study consisting of 1077 participants and is composed of five clinical variables.

The FINDRISC, Swiss Diabetes Association Score, Kahn (C and CB), Wilson, and Balkau scores are based on a sum of allocated number of points per variable. For the FINDRISC score and the SDAS, nutritional variables and familial history of diabetes for second-degree parents were not available in our cohort at baseline; thus, the threshold was reduced by 1 point. The Griffin score uses a regression equation to calculate the probability of developing T2DM. As no threshold had been proposed in the original study, a 37% probability was used to identify high-risk individuals, as proposed elsewhere [15].

Regarding ethnicity, the Wilson risk score was developed in a sample of 99% white and non-Hispanic subjects, and the Kahn scores were developed in a sample comprising 22% of black people, while no information regarding ethnicity was provided in the FINDRISC, Griffin, and Balkau scores.

#### Exclusion criteria

Eligible participants were excluded if: (1) they had no AC data; (2) they had no glucose or HbA_1_c data; (3) the covariates needed for adjustment were lacking.

## Statistical analyses

Statistical analysis was conducted using Stata version 18.0 (Stata Corp, College Station, TX, USA). Summary statistics are reported as mean ± standard deviation or as median [interquartile range] for continuous variables and as number of participants (percentage) for categorical variables. Bivariate between-group comparisons were performed using t-test or Kruskal-Wallis test for continuous variables and chi-square for categorical variables.

Bivariate associations between and incidence of diabetes (by FPG and by HbA1c) at 10-year follow up and the baseline acylcarnitine levels were computed using Wilcoxon rank-sum test. The associations were further assessed using multivariable ANOVA models adjusting on sex, age (continuous), smoking status (never, former, current), presence of a diet (yes, no), and sedentary status (yes, no). Results were presented as model-adjusted means of baseline acylcarnitine concentrations with incidence of diabetes at 10-year follow-up.

Logistic regression models were used to evaluate the association between the seven risk scores and the risk of T2DM during the follow-up period. Each individual AC was included in the models to evaluate its effect on the predictive ability of risk scores. We used logistic regression instead of Cox regression because the exact timing of diabetes onset was often uncertain, making time-to-event analyses less reliable. The diagnostic capacity of the different risk scores (with and without the individual AC species) was assessed by the AUC (area under the ROC [receiver operating characteristic] curve) and corresponding 95% CI. A comparison of the AUCs between the models with and without each AC within each risk model was performed using the roccomp command of Stata.

For sensitivity analysis, a broader endpoint of dysglycaemia was defined that included both incident diabetes and prediabetes, the latter defined as HbA1c 39–47 mmol/mol or fasting plasma glucose 5.6–6.9 mmol/L [20].

## Results

### Characteristics of the participants

Out of the 5,064 participants who attended the first follow-up of the CoLaus study during the period 2019 − 2012, 2,488 participants were ineligible, and a further 600 participants were excluded due to a lack of data on either diabetes markers or the covariates needed for adjustment, leaving 1,976 participants for the analysis (Supplementary Fig. 1). Bivariate analysis between the excluded and included participants was summarized in the Supplementary Table 2. Compared to the excluded participants, the included participants were fewer male participants, current smokers, lower BMI and waist circumference, and FPG.

After a median follow-up time of 9.1 years, 35 participants (1.8%) developed diabetes; the general characteristics at baseline of participants who developed and who did not develop diabetes are summarized in Table 1. Compared to non-diabetes participants, diabetes participants were more male participants, had higher abdominal obesity, BMI, waist circumference, SBP, DBP and FPG levels and had higher diabetes risk scores.

**Table 1:** Characteristics of the participants at baseline, by diabetes incidence at follow-up, CoLaus|PsyCoLaus study, Lausanne, Switzerland

	Non-diabetes	Diabetes	*p*-value
	(*n* = 1,941)	(*n* = 35)	
Male	792 (40.8)	23 (65.7)	**< 0.05**
Age (years)	53.3 ± 8.6	56.0 ± 8.0	0.058
Smoking categories (%)			0.083
Never	855 (44.0)	9 (25.7)	
Former	675 (34.8)	15 (42.9)	
Current	411 (21.2)	11 (31.4)	
Weekly alcohol units	6.0 ± 7.2	6.6 ± 6.8	0.61
Sedentary (%)	960 (49.5)	19 (54.3)	0.57
Body mass index (kg/m^2^)	23.95 ± 2.88	26.44 ± 2.87	**< 0.001**
Waist (cm)	85.5 ± 9.7	97.2 ± 9.2	**< 0.001**
Abdominal obesity (%)	376 (19.4)	18 (51.4)	**< 0.001**
Hypertension (%)	281 (14.5)	10 (28.6)	**< 0.05**
Systolic blood pressure (mmHg)	120 ± 16	134 ± 23	**< 0.001**
Dyastolic blood pressure (mmHg)	76 ± 10	81 ± 12	**< 0.05**
Fasting plasma glucose (mmol/L)	5.51 ± 0.49	6.16 ± 0.51	**< 0.001**
Follow-up duration (years)	9.1 (9.0–9.3)	9.0 (9.0–9.2)	0.47
Diabetes risk scores	4.5 ± 3.6	7.8 ± 6.2	**< 0.001**
Wilson score	9.1 ± 10.4	22.6 ± 16.9	**< 0.001**
Griffin score	22.1 ± 13.9	40.6 ± 15.0	**< 0.001**
Kahn clinical score	22.7 ± 14.8	46.0 ± 12.4	**< 0.001**
Kahn clinical + biology score	2.0 ± 1.1	3.0 ± 1.0	**< 0.001**
Balkau clinical score	8.5 ± 4.6	13.9 ± 3.4	**< 0.001**
Swiss Diabetes Association score	7.3 ± 4.0	11.5 ± 2.9	**< 0.001**
FINRISK score	792 (40.8)	23 (65.7)	**< 0.05**

Results are expressed as average ± standard deviation or median [interquartile range] for continuous variables and as sample size and (column percentage) for categorical variables. Between-group comparisons performed using chi-square for categorical variables and t-test for continuous variables

### Association of circulating acylcarnitine species and 10-year diabetes incidence

The bivariate and multivariate associations between the circulating AC species and 10-year incidence of diabetes are shown in Table 2. In bivariate analysis, SCAC (Propionyl- and Isovaleryl-) and MCAC (Hexanoyl- and Octenoyl-) were associated with diabetes incidence. In contrast, no LCAC showed any significant associations with diabetes incidence. The findings were further examined using multivariate analysis adjusted for the potential confounders (Table 3). SCAC (Propionyl-) remained associated with diabetes incidence, albeit there were no significant associations between diabetes incidence and MCAC or LCAC.

**Table 2:** Bivariate and multivariate associations between circulating acylcarnitine species and incidence of diabetes, CoLaus|PsyCoLaus study, Lausanne, Switzerland

		Bivariate			Multivariate		
		Non-Diabetes	Diabetes	*p*-value	Non-Diabetes	Diabetes	*p*-value
		**(** ***n*** ** = 1,941)**	**(** ***n*** ** = 35)**		**(** ***n*** ** = 1,941)**	**(** ***n*** ** = 35)**	
Short-chain	Deoxy-	752 (638–882)	829 (700–911)	0.05	765.5 ± 3.8	782.4 ± 28.5	0.56
	Acetyl-	7012 (5635–8849)	6772 (5667–8782)	0.92	7561.4 ± 61.3	7394.4 ± 461.5	0.72
	Propionyl-	360 (286–441)	405 (307–617)	**0.01**	376.3 ± 2.7	419.1 ± 20.4	**0.04**
	Butyryl-	161 (126–210)	167 (142–242)	0.16	180.1 ± 2.2	196.2 ± 16.9	0.34
	Hydroxybutyryl-	25 (17–38)	30 (20–42)	0.10	32.2 ± 0.6	33.7 ± 4.5	0.75
	Glutaryl-	48 (37–61)	48 (33–63)	0.78	50.8 ± 0.4	46.2 ± 3.4	0.18
	Hydroxyvaleryl-	21 (17–26)	24 (19–28)	0.11	22.5 ± 0.2	23.1 ± 1.6	0.71
	Isovaleryl-	84 (66–108)	107 (82–129)	**< 0.001**	90.7 ± 0.8	97.2 ± 6	0.28
	Tiglyl-	11 (8–15)	13 (9–17)	0.14	12.2 ± 0.1	12.5 ± 1	0.74
Medium-chain	Adipoyl-	19 (15–27)	17 (13–22)	0.064	22.3 ± 0.3	18.4 ± 2.3	0.09
	Hexanoyl-	34 (27–44)	40 (31–48)	**0.02**	38.3 ± 0.8	39.1 ± 6.2	0.90
	Octanoyl-	105 (78–145)	111 (81–136)	0.87	126.2 ± 3.7	118.3 ± 27.9	0.78
	Octenoyl-	61 (41–89)	70 (57–101)	**0.03**	72.7 ± 1.1	75 ± 8	0.78
	Decanoyl-	176 (128–246)	177 (120–226)	0.78	209.7 ± 5.3	193.1 ± 39.8	0.68
	Decenoyl-	70 (53–95)	67 (54–95)	0.99	77.3 ± 0.9	76.3 ± 6.4	0.87
	Lauroyl-	53 (39–74)	59 (39–71)	0.74	61 ± 1	59.1 ± 7.8	0.81
	Hydroxydodecanoyl-	11 (7–15)	12 (8–16)	0.33	12.1 ± 0.2	12.1 ± 1.1	0.97
	Dodecenoyl-	98 (75–126)	101 (81–120)	0.82	106.2 ± 1.2	105.7 ± 8.9	0.95
Long-chain	Myristoly-	24 (19–31)	26 (19–33)	0.37	26.1 ± 0.3	26.4 ± 2	0.87
	Hydroxytetradecanoyl-	5 (3–7)	6 (3–7)	0.42	5.9 ± 0.1	5.1 ± 0.7	0.28
	Tetradecenoyl-	64 (46–88)	58 (49–80)	0.64	72.6 ± 0.9	70.4 ± 7.1	0.76
	Tetradecanedienoyl-	44 (32–60)	43 (33–58)	0.96	49.2 ± 0.6	49.2 ± 4.6	0.99
	Palmitoyl-	115 (97–136)	127 (101–143)	0.11	118 ± 0.7	118.9 ± 4.9	0.85
	Hexadecenoyl-	28 (21–36)	26 (21–35)	0.86	30.2 ± 0.3	29.4 ± 2.2	0.73
	Heptadecanoyl-	4 (3–6)	4 (3–5)	0.28	4.4 ± 0	3.9 ± 0.4	0.23
	Stearoyl-	47 (37–59)	44 (34–56)	0.30	49.2 ± 0.4	43.9 ± 2.8	0.06
	Oleoyl-	172 (137–214)	166 (145–224)	0.73	182.5 ± 1.5	178.7 ± 11.1	0.73
	Octadecadienoyl-	47 (38–61)	47 (35–63)	0.97	55.4 ± 0.8	53 ± 6	0.69
	Arachidonyl-	5 (3–7)	5 (4–7)	0.28	5.4 ± 0.1	5.2 ± 0.5	0.72
	Free carnitine	752 (638–882)	829 (700–911)	0.05	34.6 ± 0.2	35.7 ± 1.2	0.39

For bivariate analysis, values are expressed as median [interquartile range] of baseline acyl- concentrations for circulating acyl- levels and between group comparisons performed using Wilcoxon rank sum test. For multivariate analysis, values are expressed as model-adjusted means of baseline acylcarnitine concentrations ± standard errors with incidence of diabetes and pre-diabetes at 10-year follow-up. Statistical analysis by multivariable ANOVA models adjusting on sex (male, female), age (continuous), smoking status (never, former, current), presence of a diet (yes, no), and sedentary status (yes, no)

**Table 3:** Predictive capacity regarding incidence of diabetes of different diabetes risk scores alone or including specific acylcarnitine species

	Odds ratio	*P*-value		AUC	*P*-value
**Wilson score**			Wilson score	0.768 (0.695–0.842)	
Propionyl-	1.015 (1.005–1.025)	**0.002**	Propionyl-	0.720 (0.633–0.807)	0.266
Hydroxyvaleryl-	1.055 (0.917–1.214)	0.453			
Isovaleryl-	1.032 (1.002–1.064)	**0.037**	Isovaleryl-	0.773 (0.709–0.836)	0.877
Tiglyl-	1.013 (1.005–1.021)	0.428			
**Griffin score**			**Griffin score**	0.828 (0.774–0.881)	
Propionyl-	1.014 (1.005–1.024)	**0.004**	Propionyl-	0.817 (0.754–0.880)	0.609
Hydroxyvaleryl-	1.06 (0.915–1.229)	0.437			
Isovaleryl-	1.03 (0.999–1.063)	0.061	Isovaleryl-	0.833 (0.783–0.883)	0.661
Tiglyl-	1.083 (0.847–1.384)	0.527			
**Kahn score1**			**Kahn score1**	0.815 (0.738–0.891)	
Propionyl-	1.013 (1.003–1.024)	**0.009**	Propionyl-	0.821 (0.745–0.898)	0.570
Hydroxyvaleryl-	1.076 (0.935–1.238)	0.307			
Isovaleryl-	1.021 (0.991–1.053)	0.172	Isovaleryl-	0.821 (0.746–0.895)	0.118
Tiglyl-	1.140 (0.886–1.465)	0.308			
**Kahn score2**			**Kahn score2**	0.875 (0.819–0.930)	
Propionyl-	1.008 (0.998–1.019)	0.13	Propionyl-	0.878 (0.823–0.933)	0.477
Hydroxyvaleryl-	1.01 (0.846–1.205)	0.916			
Isovaleryl-	1.013 (0.978–1.05)	0.476	Isovaleryl-	0.877 (0.823–0.931)	0.210
Tiglyl-	1.019 (0.785–1.323)	0.888			
**Balkau score**			**Balkau score**	0.754 (0.676–0.832)	
Propionyl-	1.015 (1.005–1.024)	**0.002**	Propionyl-	0.778 (0.702–0.854)	0.238
Hydroxyvaleryl-	1.078 (0.948–1.226)	0.253			
Isovaleryl-	1.035 (1.006–1.064)	**0.017**	Isovaleryl-	0.785 (0.710–0.859)	**0.006**
Tiglyl-	1.186 (0.925–1.519)	0.178			
**Swiss Diabetes Association Score**			**Swiss Diabetes Association Score**	0.825 (0.771–0.879)	
Propionyl-	1.015 (1.005–1.025)	**0.002**	Propionyl-	0.843 (0.788–0.897)	0.177
Hydroxyvaleryl-	1.058 (0.897–1.248)	0.501			
Isovaleryl-	1.034 (1.004–1.064)	**0.027**	Isovaleryl-	0.841 (0.789–0.892)	**0.011**
Tiglyl-	1.075 (0.831–1.390)	0.582			
**FINRISK score**			**FINRISK score**	0.794 (0.735–0.853)	
Propionyl-	1.013 (1.004–1.023)	**0.007**	Propionyl-	0.843 (0.788–0.897)	0.197
Hydroxyvaleryl-	1.055 (0.902–1.234)	0.506			
Isovaleryl-	1.035 (1.004–1.067)	**0.028**	Isovaleryl-	0.841 (0.789–0.892)	**0.023**
Tiglyl-	1.078 (0.837–1.388)	0.561			

Results are expressed as odds ratio and area under the curve (AUC), each with their 95% confidence intervals. Results for the risk score are given for a one-unit increase of the risk score. Results for acylcarnitines are given for a 5-nmol increase in blood levels, adjusting for the corresponding diabetes risk score

Sensitivity analyses using both diabetes and prediabetes incidence and diabetes incidence defined by fasting plasma glucose showed a similar pattern of associations compared to the primary analysis (Supplementary Tables 3 & Supplementary Table 4). In the multivariate analysis, SCACs remained consistently associated across all definitions with additional association with MCAC (Adipoyl-) when incident diabetes was defined by fasting plasma glucose.

### Predictive capacity of acylcarnitine added to diabetes risk scores

Logistic regression models were used to assess whether specific AC species predicted diabetes incidence after adjusting for each of the seven established diabetes risk scores (Table 3**).** Among SCAC, Propionyl- and Isovaleryl- were associated with higher odds of diabetes incidences, particularly when adjusting for the Wilson, Balkau, Swiss Diabetes Association Score and FINRISK scores. In contrast, only Propionyl- was associated with higher odds of diabetes incidence when adjusting for Griffin and Kahn scores.

**Fig. 1:**
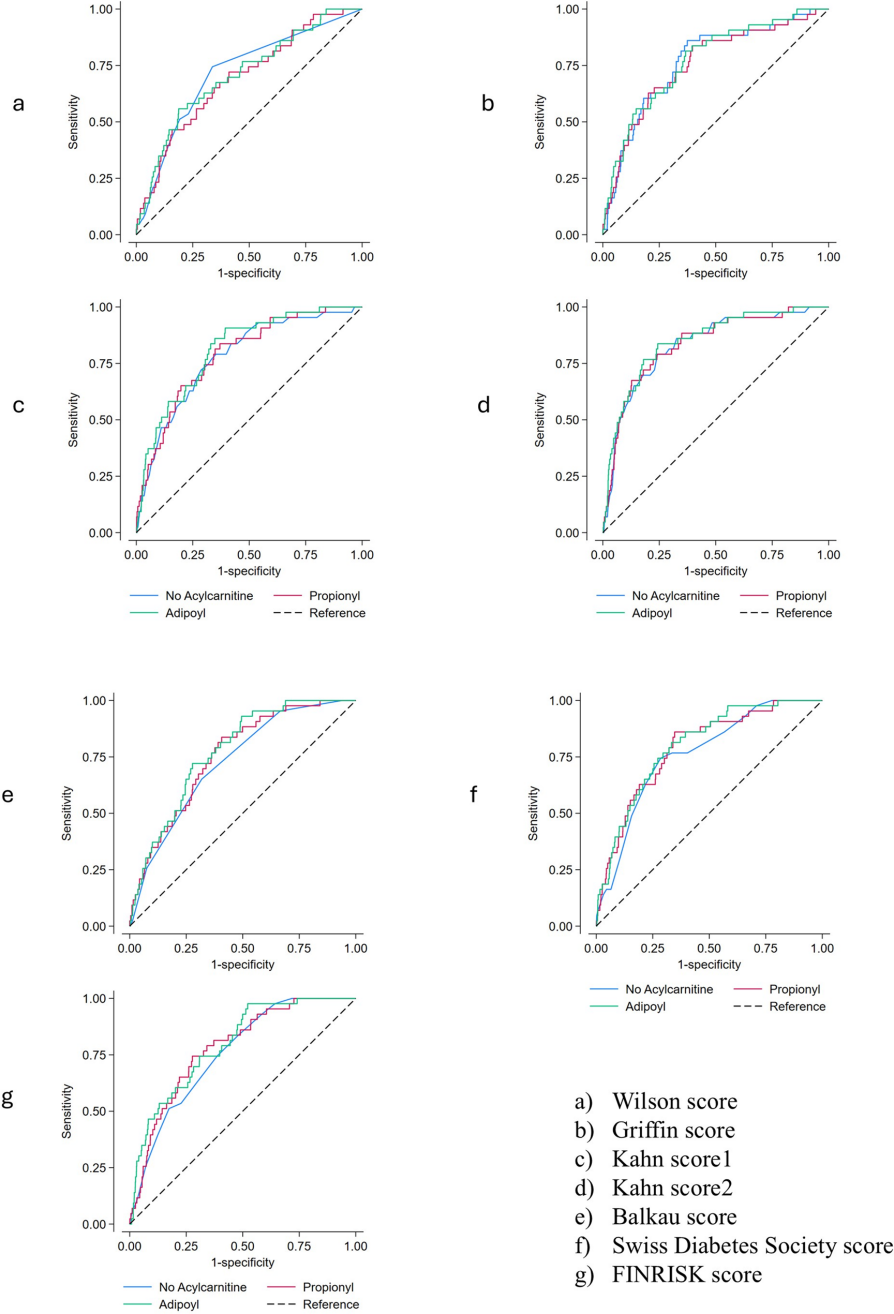
Areas under the receiver operating characteristic (ROC) curves for seven established diabetes risk scores, shown without Acylcarnitine and after addition of Adipoyl- and Propionylcarnitine

To evaluate whether these ACs improved the predictive performance of the risk scores, the AUC was calculated for each risk score (Fig. 1; Table 3**).** Adding Isovaleryl- improved AUC (≈ 0.01–0.03) in all risk scores, where the improvements in Balkau, Swiss Diabetes Association and FINRISK scores were significant. In contrast, inclusion of Propionyl- did not improve discrimination for any of the risk scores.

In descriptive analysis, the diagnostic accuracy of the risk scores was calculated with and without including Isovalerylcarnitine (Supplementary Table 5). Overall, the addition of Isovalerylcarnitine resulted in increased specificity and positive predictive value while sensitivity and negative predictive value remained unchanged.

## Discussion

To our knowledge, no prior study has exclusively examined metabolically healthy (without obesity, diabetes or cardiovascular disease) adults when assessing the associations of circulating AC species and diabetes risk. In this population-based sample of apparently healthy adults, we found that the baseline levels of SCACs were prospectively associated with the 10-year incidence of diabetes. The associations were consistent particularly in Propionyl- and Isovaleryl- after model adjustment of the established diabetes risk scores. Adding Isovaleryl- improved the diabetes risk scores, particularly Balkau, Swiss Diabetes Association and FINRISK scores. Overall, the participants who developed diabetes had higher baseline levels of circulating AC species compared to those who remained diabetes free.

### Association of circulating acylcarnitine species and 10-year diabetes and prediabetes incidence

In contrast to the previous studies [21–23] which primarily reported LCACs being associated with insulin resistance and diabetes risk, our study found a different pattern. Our results showed that SCACs were associated with diabetes incidence over 10 years and the associations were preserved in the sensitivity analyses using a broader dysglycaemia outcome (i.e., both prediabetes and diabetes). This is in line with the findings reported in previous studies [11, 21, 24]. Notably, the SCACs associated with the diabetes risk in our study were metabolites of branched‑chain amino acid (BCAA) which has previously been shown to be significantly associated with diabetes incidence. The accumulation of SCAC in the circulation may therefore reflect mitochondrial overload, impaired fatty acid oxidation and the early stage of insulin resistance [9].

In contrast, we did not observe any associations between LCAC or MCAC with diabetes incidence despite ample evidence linking the elevated circulating LCAC levels and insulin-resistant states [25, 26]. One possible explanation may be the metabolically healthy profile of our study which differs from high-risk populations as in the previous studies, consequently accounting for the difference in the LCAC-related signals. Another factor could be the strong influence of insulin on circulating LCAC levels [9]. Since insulin levels are significantly lower in the fasted state than the fed state, the circulating AC levels measured at the fasted state, as our study did, may not accurately reflect dynamic changes of LCAC levels in the postprandial state associated with insulin resistance, particularly in apparently healthy individuals. Alternatively, changes of LCAC concentrations in the fasting state may be too subtle to be detected, given that their circulating levels are much lower compared to SCAC [9].

### Predictive capacity of acylcarnitine on diabetes risk scores

Only a limited number of studies have examined the predictive capacity of ACs in apparently metabolically healthy adults, and the existing studies have mixed findings [11, 25, 27]. Importantly, no study has assessed the predictive performance of AC across several established diabetes risk scores. In this study, we observed that SCACs remained independently associated with a higher risk of diabetes beyond the traditional risk scores which agrees with another study conducted in a Mediterranean population [11]. Furthermore, adding AC to the risk scores improved the predictive performance, which agrees with the previous findings [25, 27] although contrary to the study conducted in a Mediterranean population which showed no incremental value [11]. The discrepancies between the other studies likely reflect methodology and biological differences. Previous studies often used penalised regression or metabolite-weighted scores rather than assessing individual AC species within established risk models, which limits the comparability. Furthermore, our cohort consists of metabolically healthy adults at baseline, free of obesity, diabetes and cardiovascular disease where the other cohorts included individuals with metabolic risk where AC concentrations were likely to be more prominent, potentially leading to stronger or different predictive signals.

### Strength and limitations

This study has several strengths. Precise and quantitative characterization of the AC species [28]. Another strength is the prospective design, longer follow-up and the use of multiple validated risk scores, which are methodological advantages. Moreover, inclusion of apparently metabolically healthy adults allowed us to examine the associations between AC and diabetes incidence without the additional confounding effect of insulin resistance, underlying cardiovascular disease or low-grade chronic inflammation states in the case of obesity. Furthermore, to our knowledge, this is the first study to examine the associations of circulating AC and diabetes incidence and the predictive capacity of circulating AC across several established diabetes risk scores in an apparently healthy adult population-based sample.

However, our study also has several limitations. First, the number of diabetes incidence was relatively small (*n* = 35 incidence), although the sensitivity analysis using a broader definition of dysglycaemia preserved similar findings. Second, only baseline AC levels were available, which limits the assessment of temporal changes of circulating AC levels. Third, the use of logistic regression model instead of Cox regression could result in loss of time-to-event information and potential information bias as incidence date would correspond to the date of diagnosis and not to the real date of occurrence. However, due to the uncertainty of the exact timing of diabetes onset, Cox regression could result in misclassification of the time-to-event. Moreover, no correction for multiple testing was applied, as this was an exploratory study, and we wanted to examine whether the results applied to a wide range of diabetes risk scores. Fourth, gut microbiota can modify blood levels of short AC [29], but no stools were collected during this follow-up. Fifth, the study included only very healthy participants, devoid of most metabolic risk factors (no obesity, no hypolipidemic drug treatment), which explains the low incidence of diabetes. Still, it is of interest to note that, even in this very healthy cohort, the diabetes risk scores and ACs were associated with the risk of developing diabetes. Finally, the study was conducted in Switzerland, in a sample with a relatively homogenous ethnic background, which may restrict generalizability to other geographic regions or ethnic groups.

## Conclusion

In this study of apparently healthy adults, elevated baseline levels of SCAC were consistently associated with future diabetes risk and provided addition predictive value beyond tradition risk scores. Adding selected AC to the risk scores improved the discrimination. Our findings highlight the potential role of AC as early biomarkers of diabetes. 

## Supplementary Information

Below is the link to the electronic supplementary material.

Supplementary Material 1

Supplementary Material 2

## Data Availability

The data of CoLaus|PsyCoLaus study used in this article cannot be fully shared as they contain potentially sensitive personal information on participants. According to the Ethics Committee for Research of the Canton of Vaud, sharing these data would be a violation of the Swiss legislation with respect to privacy protection. However, coded individual-level data that do not allow researchers to identify participants are available upon request to researchers who meet the criteria for data sharing of the CoLaus|PsyCoLaus Datacenter (CHUV, Lausanne, Switzerland). Any researcher affiliated to a public or private research institution who complies with the CoLaus|PsyCoLaus standards can submit a research application to [research.colaus@chuv.ch](mailto: research.colaus@chuv.ch) or [research.psycolaus@chuv.ch](mailto: research.psycolaus@chuv.ch) . Proposals requiring baseline data only, will be evaluated by the baseline (local) Scientific Committee (SC) of the CoLaus and PsyCoLaus studies. Proposals requiring follow-up data will be evaluated by the follow-up (multicentric) SC of the CoLaus|PsyCoLaus cohort study. Detailed instructions for gaining access to the CoLaus|PsyCoLaus data used in this study are available at [https://www.colaus-psycolaus.ch/professionals/how-to-collaborate/](https:/www.colaus-psycolaus.ch/professionals/how-to-collaborate) .

## Abbreviations

AC: Acylcarnitines
ACN: Acetonitrile
BCAA: Branched‑chain amino acid
BP: Blood pressure
CB: Clinical-biological risk score
DESIR: Data from the Epidemiological Study on the Insulin Resistance Syndrome
FPG: Fasting plasma glucose
FU: Follow-up
HbA1c: Haemoglobin A1c
HESI: Heated electrospray ionization
HPCL: High performance liquid chromatography
LC-HRMS: Liquid Chromatography-High Resolution Mass Spectrometry Analysis
LC-MS: Liquid Chromatography-Mass Spectrometry Analysis
LCAC: Long-chain Acylcarnitine
MCAC: Medium-chain Acylcarnitine
PKB: Protein kinase B
PTP1B: Protein tyrosine phosphatase 1B
SCAC: Short-chain Acylcarnitine
UHPLC: Ultrahigh-performance liquid chromatography
