# Supplementary figures and images for: Association of acylcarnitine species and diabetes incidence in a population-based apparently healthy cohort
Source: Cardiovasc Diabetol Endocrinol Rep. 2026 Jun 1;12:27. doi: 10.1186/s40842-026-00298-0 (PMC13224560; doi:10.1186/s40842-026-00298-0)

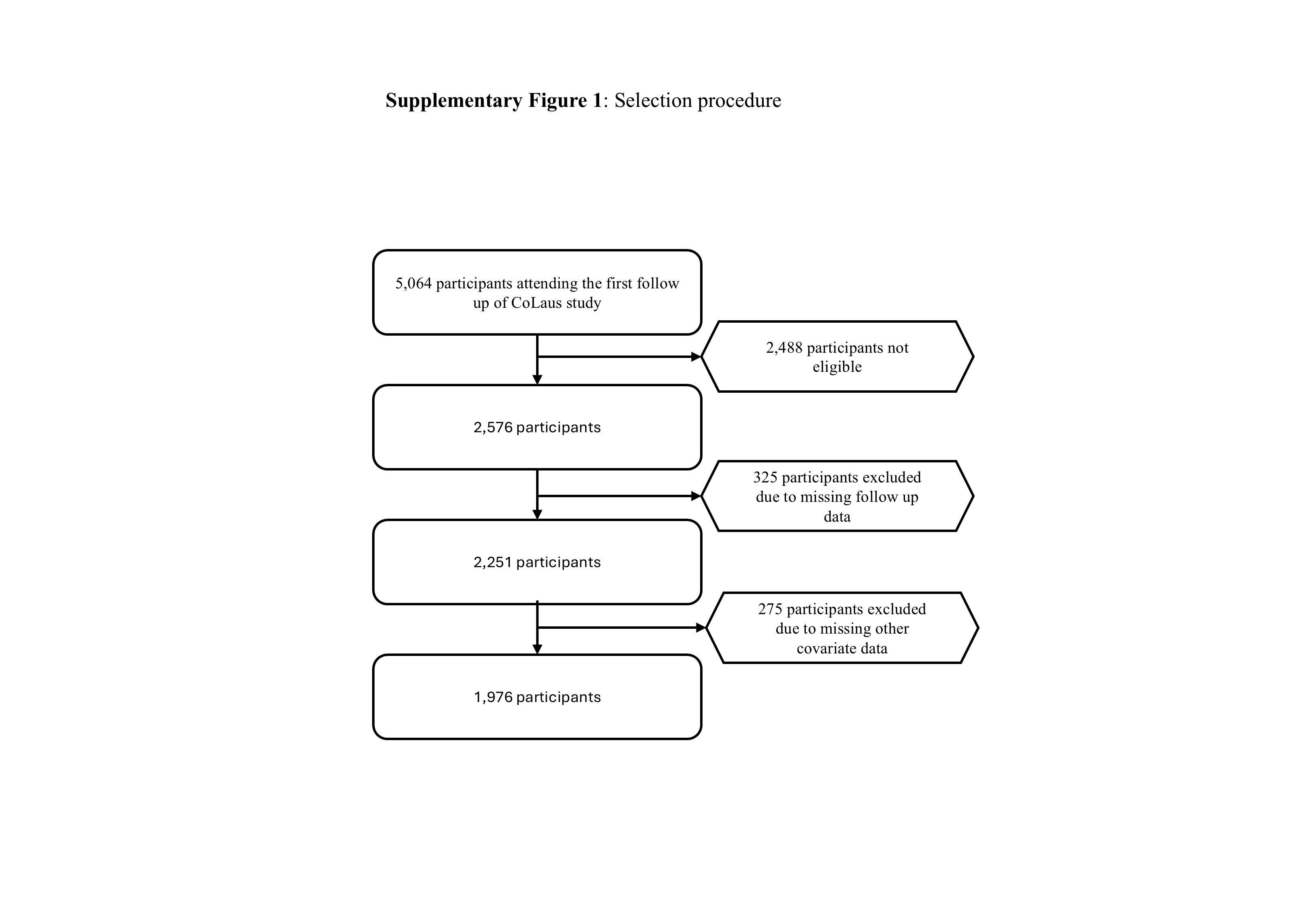

Supplement: Supplementary file 1 — Supplementary Material 1 [file 40842_2026_298_MOESM1_ESM.jpg]
