# Supplementary material for: Association of acylcarnitine species and diabetes incidence in a population-based apparently healthy cohort
Source: Cardiovasc Diabetol Endocrinol Rep. 2026 Jun 1;12:27. doi: 10.1186/s40842-026-00298-0 (PMC13224560; doi:10.1186/s40842-026-00298-0)
Supplement: Supplementary file 2 — Supplementary Material 2 [file 40842_2026_298_MOESM2_ESM.docx]

# Supplementary information

**Supplementary table 1**: Acylcarnitine species nomenclature and classification used in this study.

|  | **Acylcarnitine species (Full name)** | **Chain-length class (functional group)** |
| --- | --- | --- |
| - | Deoxy- | Precursor of carnitine |
| C2:0 | Acetyl- | Short-chain |
| C3:0 | Propionyl- | Short-chain |
| C4:0 | Butyryl- | Short-chain |
| C4-OH | Hydroxybutyryl- | Short-chain |
| C5:0-DC | Glutarylcarnitnie | Short-chain |
| C5:0-OH | Hydroxyvaleryl- | Short-chain |
| C5:0 I | Isovaleryl- | Short-chain |
| C5:1 M | Tiglyl- | Short-chain |
| C6:0-DC | Adipoyl- | Medium-chain |
| C6-OH | Hexanoyl- | Medium-chain |
| C8:0 | Octanoyl- | Medium-chain |
| C8:1 | Octenoyl- | Medium-chain |
| C10:0 | Decanoyl- | Medium-chain |
| C10:1 | Decenoyl- | Medium-chain |
| C12:0 | Lauroyl- | Medium-chain |
| C12:0 | Hydroxydodecanoyl-- | Medium-chain |
| C12:1 | Dodecenoyl-carnitne | Medium-chain |
| C14:0 | Myristolycarnitne | Long-chain |
| C14:0 | Hydroxytetradecanoyl-- | Long-chain |
| C14:1 | Tetradecenoyl-- | Long-chain |
| C14:2 | Tetradecanedienoyl-- | Long-chain |
| C16:0 | Palmitoyl- | Long-chain |
| C16:1 | Hexadecenoyl-- | Long-chain |
| C17:0 | Heptadecanoyl- | Long-chain |
| C18:0 | Stearoyl- | Long-chain |
| C18:1 | Oleoyl-- | Long-chain |
| C18:2 | Octadecadienoyl-- | Long-chain |
| C20:4 | Arachidonyl-- | Long-chain |
|  | Carnitine | Free carnitine |

Based on the References.

• Human Metabolome Database

• Dambrova M, Makrecka-Kuka M, Kuka J, Vilskersts R, Nordberg D, Attwood MM, et al. Acylcarnitines: Nomenclature, Biomarkers, Therapeutic Potential, Drug Targets, and Clinical Trials. Pharmacol Rev. 2022 Jul;74(3):506–51, <https://doi.org/10.1124/pharmrev.121.000408>

**Supplementary table 2:** Characteristics of the included and excluded participants, CoLaus|PsyCoLaus study, Lausanne, Switzerland.

|  | **Included** | **Excluded** | **p-value** |
| --- | --- | --- | --- |
|  | **(n=1,977)** | **(n=600)** |  |
| Male | 815 (41.2) | 285 (47.5) | **<0.05** |
| Age (years) | 53.3±8.6 | 53.7±8.4 | 0.29 |
| Smoking categories (%) |  |  | **<0.001** |
| Never | 865 (43.8) | 213 (37.1) |  |
| Former | 690 (34.9) | 164 (28.6) |  |
| Current | 422 (21.3) | 197 (34.3) |  |
| Weekly alcohol units | 6.0±7.2 | 6.5±9.2 | 0.21 |
| Sedentary (%) | 980 (49.6) | 143 (55.2) | 0.09 |
| Body mass index (kg/m^2^) | 23.99±2.89 | 24.36±3.05 | **<0.05** |
| Waist (cm) | 85.7±9.8 | 87.3±9.9 | **<0.001** |
| Abdominal obesity (%) | 394 (19.9) | 132 (22.1) | 0.25 |
| Hypertension (%) | 291 (14.7) | 97 (16.2) | 0.38 |
| Systolic blood pressure (mmHg) | 120±16 | 121±17 | 0.73 |
| Dyastolic blood pressure (mmHg) | 76±10 | 76±10 | 0.19 |
| Fasting plasma glucose (mmol/L) | 5.52±0.50 | 5.59±0.51 | **<0.05** |
| Diabetes risk scores | 4.6±3.7 | 5.2±4.6 | **<0.05** |
| Wilson score | 9.3±10.7 | 12.2±13.6 | **<0.001** |
| Griffin score | 22.4±14.1 | 24.7±14.2 | **<0.001** |
| Kahn clinical score | 23.1±15.1 | 26.2±15.7 | **<0.001** |
| Kahn clinical + biology score | 2.0±1.1 | 2.2±1.0 | **<0.001** |
| Balkau clinical score | 8.6±4.6 | 8.7±4.5 | 0.69 |
| Swiss Diabetes Association score | 7.4±4.0 | 7.4±3.8 | 0.94 |
| FINRISK score | 815 (41.2) | 285 (47.5) | **<0.05** |

Results are expressed as number of participants (column percentage) for categorical variables and as mean standard deviation for continuous variables. Between group comparisons performed using chi-square for categorical variables and student’s t-test) for continuous variables. Due to missing values, the numbers in the excluded column might not add to total.

**Supplementary table 3:** Bivariate and multivariate associations between circulating acylcarnitine species and incidence of diabetes and prediabetes, CoLaus|PsyCoLaus study, Lausanne, Switzerland.

|  |  | Bivariate | | | Multivariate | | |
| --- | --- | --- | --- | --- | --- | --- | --- |
|  |  | Non-Diabetes | Diabetes + Prediabetes | p- value | Non-Diabetes | Diabetes + Prediabetes | p-value |
|  |  | (n=1,092) | (n=884) |  | (n=1,092) | (n=884) |  |
| Short-chain | Deoxy- | 743 (623-868) | 767 (654-899) | **0.001** | 761.9 ± 5.1 | 770.3 ± 5.7 | 0.28 |
|  | Acetyl- | 6963 (5528-8710) | 7081 (5785-9042) | **0.030** | 7481.8 ± 82.9 | 7652.7 ± 92.7 | 0.18 |
|  | Propionyl- | 341 (277-417) | 382 (305-473) | **<0.001** | 366.9 ± 3.7 | 389.9 ± 4.1 | **<0.001** |
|  | Butyryl- | 156 (120-199) | 167 (130-225) | **<0.001** | 175.2 ± 3 | 186.8 ± 3.4 | **0.01** |
|  | Hydroxybutyryl- | 24 (17-37) | 26 (19-39) | **<0.001** | 31.7 ± 0.8 | 33 ± 0.9 | 0.32 |
|  | Glutaryl- | 48 (37-61) | 48 (38-61) | 0.30 | 50.7 ± 0.6 | 50.7 ± 0.7 | 0.94 |
|  | Hydroxyvaleryl- | 21 (16-25) | 22 (18-27) | **<0.001** | 21.6 ± 0.3 | 23.7 ± 0.3 | **<0.001** |
|  | Isovaleryl- | 79 (62-101) | 89 (71-117) | **<0.001** | 87.5 ± 1.1 | 94.8 ± 1.2 | **<0.001** |
|  | Tiglyl- | 11 (8-15) | 12 (8-16) | **<0.001** | 11.7 ± 0.2 | 12.8 ± 0.2 | **<0.001** |
| Medium-chain | Adipoyl- | 19 (14-26) | 19 (15-27) | 0.89 | 22.3 ± 0.4 | 22.2 ± 0.5 | 0.88 |
|  | Hexanoyl- | 33 (26-43) | 36 (28-45) | **<0.001** | 38.6 ± 1.1 | 38.1 ± 1.2 | 0.74 |
|  | Octanoyl- | 104 (76-145) | 106 (79-146) | 0.43 | 131.4 ± 5 | 119.9 ± 5.6 | 0.13 |
|  | Octenoyl- | 58 (40-85) | 64 (44-95) | **<0.001** | 71.7 ± 1.4 | 73.8 ± 1.6 | 0.34 |
|  | Decanoyl- | 176 (125-245) | 175 (132-245) | 1.00 | 217.5 ± 7.1 | 199.5 ± 8 | 0.10 |
|  | Decenoyl- | 68 (52-94) | 72 (54-96) | 0.14 | 77.6 ± 1.2 | 76.9 ± 1.3 | 0.70 |
|  | Lauroyl- | 53 (38-73) | 54 (40-75) | 0.24 | 62.3 ± 1.4 | 59.2 ± 1.6 | 0.15 |
|  | Hydroxydodecanoyl- | 11 (7-15) | 11 (8-16) | **0.021** | 12.1 ± 0.2 | 12.1 ± 0.2 | 0.92 |
|  | Dodecenoyl- | 96 (74-124) | 102 (78-129) | **0.008** | 105.8 ± 1.6 | 106.7 ± 1.8 | 0.73 |
| Long-chain | Myristoly- | 24 (19-31) | 25 (20-32) | **0.034** | 26.3 ± 0.4 | 25.8 ± 0.4 | 0.42 |
|  | Hydroxytetradecanoyl- | 5 (3-7) | 5 (3-8) | **<0.001** | 5.8 ± 0.1 | 5.9 ± 0.1 | 0.84 |
|  | Tetradecenoyl- | 64 (46-89) | 64 (47-87) | 0.50 | 73.8 ± 1.3 | 71.1 ± 1.4 | 0.18 |
|  | Tetradecanedienoyl- | 43 (32-60) | 44 (33-60) | 0.23 | 49.8 ± 0.8 | 48.5 ± 0.9 | 0.33 |
|  | Palmitoyl- | 114 (95-135) | 118 (99-139) | **0.001** | 117.8 ± 0.9 | 118.4 ± 1 | 0.66 |
|  | Hexadecenoyl- | 28 (21-36) | 28 (21-37) | 0.41 | 30.5 ± 0.4 | 29.8 ± 0.4 | 0.28 |
|  | Heptadecanoyl- | 4 (3-6) | 4 (3-6) | 0.73 | 4.4 ± 0.1 | 4.4 ± 0.1 | 0.63 |
|  | Stearoyl- | 47 (36-59) | 47 (37-59) | 0.35 | 49.6 ± 0.5 | 48.4 ± 0.6 | 0.12 |
|  | Oleoyl- | 172 (139-212) | 171 (136-216) | 0.42 | 182.8 ± 2 | 182.1 ± 2.2 | 0.81 |
|  | Octadecadienoyl- | 47 (37-60) | 48 (38-63) | **0.040** | 54.6 ± 1.1 | 56.3 ± 1.2 | 0.29 |
|  | Arachidonyl- | 5 (3-7) | 5 (3-7) | **0.002** | 5.3 ± 0.1 | 5.4 ± 0.1 | 0.80 |
|  | Free carnitine | 33 (29-38) | 36 (31-41) | **<0.001** | 34 ± 0.2 | 35.4 ± 0.2 | **<0.001** |

FPG: fasting plasma glucose, HbA_1_c: glycated hemoglobin. Prediabetes and diabetes were analysed as separate outcomes. For bivariate analysis, values are expressed as median [interquartile range] of baseline acyl- concentrations for circulating acyl- levels and between group comparisons performed using Wilcoxon rank sum test. For multivariate analysis, values are expressed as model-adjusted means of baseline acylcarnitine concentrations ± standard errors with incidence of diabetes and pre-diabetes at 10-year follow-up. Statistical analysis by multivariable ANOVA models adjusting on sex (male, female), age (continuous), smoking status (never, former, current), presence of a diet (yes, no), and sedentary status (yes, no).

**Supplementary table 4:** Bivariate and multivariate associations between circulating acylcarnitine species and incidence of diabetes by FPG, CoLaus|PsyCoLaus study, Lausanne, Switzerland.

|  |  | Bivariate | | | Multivariate | | |
| --- | --- | --- | --- | --- | --- | --- | --- |
|  |  | Non-Diabetes | Diabetes (FPG) | p- value | Non-Diabetes | Diabetes (FPG) | p-value |
|  |  | (n=1,933) | (n=43) |  | (n=1,933) | (n=43) |  |
| Short-chain | Deoxy- | 752 (638-883) | 783 (688-888) | 0.43 | 744.9 ± 25.7 | 766.2 ± 3.8 | 0.41 |
|  | Acetyl- | 7013 (5641-8852) | 6772 (5278-8639) | 0.44 | 7255 ± 416 | 7563.9 ± 61.4 | 0.46 |
|  | Propionyl- | 359 (285-441) | 428 (316-553) | **<0.05** | 418.8 ± 18.4 | 376.1 ± 2.7 | **<0.05** |
|  | Butyryl- | 161 (126-210) | 177 (143-242) | **<0.05** | 198.2 ± 15.2 | 180 ± 2.2 | 0.24 |
|  | Hydroxybutyryl- | 25 (17-38) | 27 (19-40) | 0.30 | 32.5 ± 4 | 32.2 ± 0.6 | 0.94 |
|  | Glutaryl- | 48 (37-61) | 44 (33-55) | 0.26 | 44.5 ± 3 | 50.9 ± 0.4 | **<0.05** |
|  | Hydroxyvaleryl- | 21 (17-26) | 23 (18-28) | 0.32 | 22.2 ± 1.4 | 22.6 ± 0.2 | 0.81 |
|  | Isovaleryl- | 84 (66-108) | 102 (81-129) | **<0.05** | 95.2 ± 5.5 | 90.7 ± 0.8 | 0.41 |
|  | Tiglyl- | 11 (8-15) | 12 (9-16) | 0.43 | 11.9 ± 0.9 | 12.2 ± 0.1 | 0.79 |
| Medium-chain | Adipoyl- | 19 (15-27) | 17 (12-21) | **<0.05** | 17.6 ± 2.1 | 22.3 ± 0.3 | **<0.05** |
|  | Hexanoyl- | 34 (27-44) | 40 (28-47) | 0.08 | 38.7 ± 5.6 | 38.3 ± 0.8 | 0.94 |
|  | Octanoyl- | 105 (78-145) | 114 (76-145) | 0.90 | 116.5 ± 25.2 | 126.3 ± 3.7 | 0.70 |
|  | Octenoyl- | 61 (41-89) | 68 (46-95) | 0.13 | 72.4 ± 7.2 | 72.7 ± 1.1 | 0.97 |
|  | Decanoyl- | 176 (128-245) | 177 (110-240) | 0.54 | 190.6 ± 35.8 | 209.8 ± 5.3 | 0.60 |
|  | Decenoyl- | 70 (53-95) | 65 (42-99) | 0.34 | 74.4 ± 5.8 | 77.3 ± 0.9 | 0.61 |
|  | Lauroyl- | 53 (39-74) | 55 (32-72) | 0.82 | 58.5 ± 7.1 | 61 ± 1 | 0.73 |
|  | Hydroxydodecanoyl- | 11 (7-15) | 11 (6-15) | 0.58 | 11 ± 1 | 12.2 ± 0.2 | 0.25 |
|  | Dodecenoyl- | 98 (75-126) | 98 (77-118) | 0.62 | 102 ± 8 | 106.3 ± 1.2 | 0.60 |
| Long-chain | Myristoly- | 24 (19-31) | 25 (18-32) | 1.00 | 25.7 ± 1.8 | 26.1 ± 0.3 | 0.82 |
|  | Hydroxytetradecanoyl- | 5 (3-7) | 5 (3-7) | 0.59 | 4.9 ± 0.6 | 5.9 ± 0.1 | 0.10 |
|  | Tetradecenoyl- | 64 (46-88) | 56 (40-88) | 0.24 | 68.6 ± 6.4 | 72.7 ± 1 | 0.53 |
|  | Tetradecanedienoyl- | 44 (32-60) | 39 (28-58) | 0.37 | 47.2 ± 4.1 | 49.3 ± 0.6 | 0.61 |
|  | Palmitoyl- | 116 (97-136) | 125 (99-143) | 0.19 | 119.7 ± 4.4 | 118 ± 0.7 | 0.70 |
|  | Hexadecenoyl- | 28 (21-36) | 26 (20-35) | 0.27 | 29 ± 2 | 30.2 ± 0.3 | 0.56 |
|  | Heptadecanoyl- | 4 (3-6) | 4 (3-5) | 0.41 | 4.2 ± 0.3 | 4.4 ± 0 | 0.60 |
|  | Stearoyl- | 47 (37-59) | 44 (35-56) | 0.30 | 45.2 ± 2.5 | 49.2 ± 0.4 | 0.11 |
|  | Oleoyl- | 172 (138-214) | 164 (135-227) | 0.90 | 178 ± 10 | 182.5 ± 1.5 | 0.66 |
|  | Octadecadienoyl- | 47 (38-61) | 47 (35-64) | 0.95 | 54.4 ± 5.4 | 55.4 ± 0.8 | 0.85 |
|  | Arachidonyl- | 5 (3-7) | 5 (4-7) | 0.34 | 5.4 ± 0.4 | 5.4 ± 0.1 | 0.96 |
|  | Free carnitine | 34 (29-39) | 36 (32-43) | 0.06 | 35.1 ± 1.1 | 34.6 ± 0.2 | 0.69 |

FPG: fasting plasma glucose. For bivariate analysis, values are expressed as median [interquartile range] of baseline acyl- concentrations for circulating acyl- levels and between group comparisons performed using Wilcoxon rank sum test. For multivariate analysis, values are expressed as model-adjusted means of baseline acylcarnitine concentrations ± standard errors with incidence of diabetes and pre-diabetes at 10-year follow-up. Statistical analysis by multivariable ANOVA models adjusting on sex (male, female), age (continuous), smoking status (never, former, current), presence of a diet (yes, no), and sedentary status (yes, no).

**Supplementary table 5:** Diagnostic accuracy of risk scores alone and risk scores including Isovalerylcarnitine.

|  | **Sensitivity** | **Specificity** | **Positive predictive value** | **Negative predictive value** |
| --- | --- | --- | --- | --- |
| **Wilson score** |  |  |  |  |
| Model 1 | 82.9 (66.4 - 93.4) | 66.3 (64.1 - 68.4) | 4.2 (2.9 – 6.0) | 99.5 (99.0 - 99.8) |
| Model 2 | 88.6 (73.3 - 96.8) | 58.8 (56.6 - 61.0) | 3.7 (2.6 - 5.3) | 99.7 (99.1 - 99.9) |
| **Griffin score** |  |  |  |  |
| Model 1 | 94.3 (80.8 - 99.3) | 63.8 (61.6 - 65.9) | 4.5 (3.1 - 6.2) | 99.8 (99.4 - 100) |
| Model 2 | 91.4 (76.9 - 98.2) | 62.3 (60.1 - 64.4) | 4.2 (2.9 - 5.9) | 99.8 (99.3 - 100) |
| **Kahn score1** |  |  |  |  |
| Model 1 | 74.3 (56.7 - 87.5) | 79.9 (78.1 - 81.7) | 6.2 (4.1 – 9.0) | 99.4 (98.9 - 99.7) |
| Model 2 | 74.3 (56.7 - 87.5) | 80.0 (78.2 - 81.8) | 6.3 (4.1 - 9.1) | 99.4 (98.9 - 99.7) |
| **Kahn score2** |  |  |  |  |
| Model 1 | 80.0 (63.1 - 91.6) | 82.1 (80.3 - 83.8) | 7.5 (5.0 - 10.6) | 99.6 (99.1 - 99.8) |
| Model 2 | 77.1 (59.9 - 89.6) | 82.3 (80.5 - 84.0) | 7.3 (4.9 - 10.4) | 99.5 (99.0 - 99.8) |
| **Balkau score** |  |  |  |  |
| Model 1 | 74.3 (56.7 - 87.5) | 68.1 (66.0 - 70.2) | 4.0 (2.7 - 5.8) | 99.3 (98.7 - 99.7) |
| Model 2 | 74.3 (56.7 - 87.5) | 69.0 (66.8 - 71.0) | 4.1 (2.7 – 6.0) | 99.3 (98.7 - 99.7) |
| **Swiss Diabetes Association** |  |  |  |  |
| Model 1 | 85.7 (69.7 - 95.2) | 71.5 (69.5 - 73.5) | 5.1 (3.5 - 7.3) | 99.6 (99.2 - 99.9) |
| Model 2 | 85.7 (69.7 - 95.2) | 73.8 (71.8 - 75.7) | 5.6 (3.8 - 7.8) | 99.7 (99.2 - 99.9) |
| **FINRISK score** |  |  |  |  |
| Model 1 | 82.9 (66.4 - 93.4) | 61.4 (59.2 - 63.6) | 3.7 (2.5 - 5.3) | 99.5 (98.9 - 99.8) |
| Model 2 | 82.9 (66.4 - 93.4) | 62.6 (60.4 - 64.7) | 3.8 (2.6 - 5.5) | 99.5 (98.9 - 99.8) |

Model 1: Risk score alone, Model 2: Risk score + Isovalerylcarnitine

**Supplementary table 6:** Description of the diabetes risk scores.

|  | **FINDRISC** | **Swiss Diabetes association** | **Wilson et al.** | **Griffin et al.** | **Balkau et al.** | **Kahn et al (C)** | **Kahn et al. (CB)** |
| --- | --- | --- | --- | --- | --- | --- | --- |
| Sample size | 4’435 |  | 3’140 | 1’077 | 3’817 | 12’729 | 12’279 |
| Follow-up time | 10 years |  | 7 years | cross-section. | 9 years | 9 years | 9 years |
| Incidence of DM | 4.1% |  | 5.1% | 4.46% | 5.61% | 19% | 19% |
| Sex |  |  |  | X | X |  |  |
| Age | X | X |  | X |  | X | X |
| Black race |  |  |  |  |  | X | X |
| Clinical data |  |  |  |  |  |  |  |
| BMI | X | X | X | X |  |  |  |
| Weight |  |  |  |  |  | X |  |
| Waist | X | X |  |  | X | X | X |
| Height |  |  |  |  |  | X | X |
| Hypertension* | M | M | A/M | M | A/M | X | X |
| Resting heart rate |  |  |  |  |  | X | X |
| Family history of diabetes |  | X | X | X | X | X | X |
| Personal history of hyperglycemia | X | X |  |  |  |  |  |
| Corticosteroids |  |  |  | X |  |  |  |
| Lifestyle data |  |  |  |  |  |  |  |
| Physical activity | X | X |  |  |  |  |  |
| Smoking |  |  |  | X | X | X |  |
| Alcohol |  |  |  |  |  |  | X |
| Fruit & vegetable consumption | X | X |  |  |  |  |  |
| Education |  |  |  |  |  | X |  |
| Blood markers |  |  |  |  |  |  |  |
| Glucose |  |  | X |  |  |  | X |
| Triglycerides |  |  | X |  |  |  | X |
| High density lipoprotein |  |  | X |  |  |  | X |
| Uric acid |  |  |  |  |  |  | X |
| Score threshold | ≥ 9 pts | ≥ 9 pts | 24 pts | 37% | ≥5 pts | ≥38 pts | ≥38 pts |
| Risk of developing T2DM | 13% | 13% | 33% | 37% | 30% | 17.7% | 17.7% |

Abbreviations: BMI, body mass index; T2DM, type 2 diabetes mellitus. *A: anamnestic, M: antihypertension medication.

Details of the diabetes risk scores derived from Kraege V et al., 2013. [10.5281/zenodo.3520776](https://zenodo.org/record/3520776).
